# Johne’s Disease Control in Beef Cattle: Balancing Test-and-Cull Strategies with Economic and Epidemiological Trade-Offs

**DOI:** 10.3390/vetsci12121210

**Published:** 2025-12-17

**Authors:** Leigh Rosengren, Steven M. Roche, Kathy Larson, Cheryl L. Waldner

**Affiliations:** 1 Large Animal Clinical Sciences, Western College of Veterinary Medicine, University of Saskatchewan, Saskatoon, SK S7N 5B4, Canada; 2 ACER Consulting Limited, Guelph, ON N1G 5L3, Canada; 3 Agricultural and Resource Economics, College of Agriculture and Bioresources, University of Saskatchewan, Saskatoon, SK S7N 5A8, Canada; kathy.larson@usask.ca

**Keywords:** Johne’s disease, test-and-cull, diagnostic testing, simulation modeling, net present value, economics, *Mycobacterium avium* spp. *paratuberculosis*

## Abstract

Johne’s disease (JD) is a chronic infection of cattle that undermines herd productivity and profitability. While test-and-cull programs are commonly proposed for control, their effectiveness and economic feasibility remain uncertain in beef production systems. This study used an updated agent-based model (ABM) to simulate JD transmission in a representative 300-cow Western Canadian beef herd, coupled with a partial budget model to evaluate net present value (NPV) over a 10-year time horizon. Seven diagnostic test-and-cull strategies were compared, varying in test type (ELISA, individual PCR, and pooled PCR), sampling frequency (6, 12, or 24 mo), and risk-based sampling protocols. Results showed that, under baseline assumptions (6% starting prevalence; 1% prevalence in purchased stock), all strategies reduced JD prevalence relative to no testing, and six of seven yielded higher NPVs. Annual individual PCR testing provided the best balance between prevalence reduction and profitability, whereas semi-annual PCR most effectively reduced prevalence but at greater economic cost. Failure to implement control measures resulted in increasing prevalence and long-term economic losses. Sensitivity analyses demonstrated that strategy performance was consistent across variations in market conditions, cost of production, and replacement female management, although profitability declined substantially when JD prevalence in externally sourced stock was high (i.e., 10%). Collectively, these findings indicate that JD can be controlled economically in beef herds, with long-term application of various test-and-cull strategies offering robust options adaptable to management preferences.

## Simple Summary

Johne’s disease (JD) is an untreatable intestinal infection in cattle that reduces herd health and profitability. To understand how well different control programs work in beef herds, we combined a computer simulation to model JD spread in a typical 300-cow Western Canadian operation with an economic model to estimate financial outcomes over 10 years. We evaluated seven test-and-cull strategies that used different diagnostic tests and testing schedules, including risk-based sampling. All strategies lowered the frequency of JD compared with doing nothing, and most improved overall profitability. Testing every year with individual PCR provided the best mix of disease reduction and financial return. Testing every six months with PCR reduced disease the most but cost more. Herds that did not use any control saw JD levels rise and experienced long-term economic losses. The results were generally stable across changes in production costs, market prices, and replacement-heifer management. However, profits dropped when purchased animals had a high JD prevalence. Overall, this study shows that JD can be economically controlled in beef herds using long-term test-and-cull programs tailored to producer goals.

## 1. Introduction

Bovine paratuberculosis, commonly known as Johne’s disease (JD), is a chronic and untreatable disease of ruminants caused by *Mycobacterium avium* spp. *paratuberculosis* (MAP) [1]. Johne’s disease is production limiting and creates economic losses due to several outcomes in sub-clinically and clinically infected animals, which include poor feed conversion, increased predisposition to other diseases, premature culling, discounted market value, infertility, and mortality [2,3,4]. The losses are insidious as the prevalence of JD rises within a herd.

Most beef herds allow animal introductions, which creates a potential infection route. Quarantine and testing before animal introduction is often not feasible as infected animals initially have a silent non-clinical and non-detectable disease phase. Once in the herd, sub-clinical shedding animals can perpetuate and spread JD within herds as beef cows have a long productive life with prolonged contact between cows and calves [1].

Recommendations to prevent and control JD commonly focus on the implementation of a test-and-cull strategy in combination with management changes [1,5]. The choice of test-and-cull strategies, however, is not always straightforward and is influenced by a given beef producer’s objective (i.e., disease control versus disease freedom in the herd). Further, selecting an optimal test and control strategy for JD is complicated because it must balance up-front costs against long-term losses while accounting for biological uncertainty and market variability [3,6]. The silent non-clinical disease phase is followed by sub-clinical disease with intermittent shedding. At this stage, diagnostic tests can identify animals but with imperfect sensitivity [7,8]. Intermittent shedding and imperfect test performance complicate the design of effective test-and-cull strategies, and an effective testing strategy must be multi-year [1].

Producers may be motivated to test and cull for JD to improve health, financial gain, or both. Importantly, these decisions would be meaningfully informed with insight into the net economic effects of a long-run test-and-cull strategy, which accounts for the direct costs from testing and the indirect costs associated with culling non-clinical cows, and ideally insight that demonstrates that the costs associated with implementation of a test-and-cull strategy are less than the projected losses attributable to JD. Modeling the effect of test-and-cull strategies on disease prevalence and profitability creates a transparent way for producers to balance these two potentially conflicting goals [3,5]. A well accepted economic indicator used to guide decisions is net present value (NPV), which recognizes the time value of money (with the value of money today being more than the value of money in the future) and is calculated as the sum of all future net cash flows to determine the present value of a decision or investment in present day dollars [9].

Johne’s disease has long been ranked as a prevalent condition and among the costliest contagious diseases in dairy [10]. Though the within-herd prevalence of MAP within Canadian cow–calf herds has been previously reported as low (~1%), herd level data based on a subset of cows showed that 24% of herds had at least one positive animal and 5% had at least two positive animals [11]. The issue is particularly pressing in Western Canada as smaller herds are exiting the industry and dispersing their breeding stock to other herds [12]. This attrition in small herds has led to fewer, larger herds that increase their size through the purchase of new animals or the amalgamation of smaller herds with unknown MAP status, which presents risk for introduction of MAP [13].

The primary objective of this study was to evaluate test-and-cull strategies for Johne’s disease in Western Canadian beef herds using an existing stochastic simulation model. Specifically, strategies were assessed against three criteria: (i) feasibility within typical animal handling patterns, (ii) effectiveness in maintaining or reducing JD prevalence at 10 years, and (iii) profitability compared with no testing, as measured by the 10-year NPV. A secondary objective was to quantify the relative economic cost of Johne’s disease to the beef industry and to assess the value of maintaining JD-free herds under different replacement and market scenarios.

## 2. Materials and Methods

### 2.1. Agent-Based Model

The present study leveraged a previously reported agent-based model (ABM) that described JD transmission in a 300-head breeding cow herd reflective of management practices common in cow–calf operations in Western Canada [14]. The objective of the model was to compare the relative effectiveness of different diagnostic testing, culling, and replacement scenarios for the control of JD. The population, infection dynamics, and diagnostic test performance parameters had been reported in detail [14], following the Overview, Design concepts, and Details (ODD) protocol for describing individual-based and ABMs [15,16]. An updated version of this model provided the necessary stochastic outputs to serve as the basis for economic modeling in the present study. Updates to the model used to generate the data in the economic analyses, including parameter values, were also documented as per the ODD protocol and are provided in supplemental materials (Supplemental File S1); a working copy of the model is available through a basic interface at https://www.beefresearch.ca/tools/johnes-disease-calculator/ (accessed on 25 October 2025).

In the baseline scenario for the ABM, each iteration began with a 6% JD prevalence in the breeding herd [14]. An internal replacement female management choice was used, which involved retaining (i.e., not selling) heifer calves at a rate that was projected to maintain the breeding herd. Mature breeding females were purchased only when, due to variation in the number of heifers bred or mature cows culled, the retained heifers were insufficient to maintain the breeding population at 300. Bred heifers were sold to maintain herd size when excess heifers had been retained. The baseline JD prevalence in mature bred cows available for purchase was assumed to be 1%. All breeding stock transactions occurred at the start of the “winter” season to correspond to the typical practice of fall weaning and pregnancy detection.

Calibrated values were estimated for the current version of the model and model parameters (Supplemental File S1). Automated calibration experiments using the widely implemented OptQuest global optimization routine were created in the commercial software program (AnyLogic 8 Professional version 8.9.3, AnyLogic North America, Oakbrook Terrace, IL, USA) to identify the value of the parameter for the frequency of infective contact for fecal transmission of MAP and the probability of dam-to-calf transmission from birth to weaning (Supplemental File S1). The model was calibrated using regional prevalence data from surveillance initiatives and control programs with longitudinal data for de-identified individual infected herds. Updated conservative sensitivity and specificity estimates for the fecal PCR test and the serum ELISA test were modeled with PERT distributions to reflect the uncertainty in test performance with distribution parameters updated from Johnson et. al. [8,14]. Parameter values varied by test (PCR or ELISA), whether the fecal samples were tested individually or pooled, and the stage of infection (Supplementary Materials S1). All test positive animals were assumed to be culled from the herd.

Annual and semi-annual outputs for population dynamics, weaning weights of calves, testing activities, test results, and disease prevalence were generated for each iteration of the ABM. Population dynamics included all events that related to animal inventory changes, including births, deaths, sales, and purchases, stratified as appropriate by weight, age, gender, and body condition [14]. The simulated results for each iteration (*n* = 5000) were used as the inputs for the subsequent economic analysis for each management scenario (see Section 2.3, Section 2.5 and Section 2.6).

The resulting output of the ABM also provided simulated estimates of 10-year JD prevalence for each iteration (*n* = 5000) for each test-and-cull scenario examined in this investigation (see Section 2.3, Section 2.5 and Section 2.6).

### 2.2. Partial Budget and Net Present Value Model

Outputs from the ABM were applied to a partial budget, which was developed to estimate annual net profits associated with livestock sales, livestock purchases, JD sampling and testing, and variable expenses for inputs affected by the population present. The ABM data describing annual and semi-annual population dynamics, testing activity, test results, and disease prevalence were exported from AnyLogic and imported into a commercial software program (Stata/SE16, StataCorp LLC, College Station, TX, USA).

Revenue and expense (economic) parameters in the partial budget related to livestock sales and purchases were representative of Saskatchewan, Canada, and were estimated as the product of head and market price for breeding stock and the product of the liveweight and market price for all other transactions. Revenues were generated exclusively from livestock sales. Revenues accrued from the sale of weaned calves, culled mature breeding stock, non-pregnant yearling heifers, and excess bred heifers. The primary livestock market values and expense parameters included in the partial budget are described below (see Supplemental Files S2 and S3 for additional details). The final output was summarized as annual cash flow, reported in CAD.

#### 2.2.1. Livestock Market Values

Cattle in the ABM were divided into seven livestock classes based on gender, age, and production purpose, each with an average live weight and animal unit equivalent, calculated as the live animal weight (in pounds) divided by 1000 and raised to the 0.75 power. Livestock market prices were derived from five-year averages (2017–2021) reported by the Saskatchewan Crop Insurance Corporation (SCIC) for October through December, reflecting the key marketing period in Western Canada. Prices for feeder animals were based on 100 lb (45.4 kg) weight classes, using the 501–600 lb (227–272 kg) category (average 550 lb (249 kg)) for both steer and heifer calves, with a CAD 0.10/hundred lbs of weight (cwt) price slide applied to adjust for weight differences. Open yearling heifers were valued using the 901–1000 lb (409–454 kg) class, while bred heifers were priced per head as reported by SCIC (Supplementary Materials S2).

Cull cows were categorized by age and body condition, with “good” cows defined as having a body condition score (BCS) of 2.5 or higher and “thin” cows (BCS < 2.5) assumed to be 200 lbs (91 kg) lighter. Eighty percent of thin cows were assumed to have market value, while 20% were unmarketable and assumed to have zero market value. Young cows in good condition (BCS 2.5 or greater) were valued at D1/D2 market prices, while young cows that were thin and all older cows were discounted (by approximately 10%; Supplementary Materials S2 and S3) to reflect D3 prices. Mature bred cows and market bulls were valued based on reported per-head or monthly market data. Annual livestock revenue was calculated as the sum of revenues from all breeding, production, and market classes, determined by multiplying either the number of head or live weight by the appropriate market price.

#### 2.2.2. Expenses

Expenses arose from three categories: breeding stock purchases, JD sampling and testing, and variable inputs from feed, veterinary costs, and labor.

Breeding stock expenses were the product of head purchased and market price. The cost of JD testing was estimated for sample collection and diagnostic testing. A CAD 5 per head sample collection fee was set for blood collection and CAD 2.50 per head for fecal sample collection [14]. This fee included costs for professional time to collect, prepare, and ship samples as well as consumables used in sample collection. Courier costs to submit samples were estimated at CAD 25 per visit. Strategies with annual sampling in the fall did not include fees for mileage or professional veterinary services beyond sample collection, as it was assumed the veterinarian was attending the herd for pregnancy detection. Strategies with semi-annual sampling included an estimated minimal mileage fee of CAD 80 and veterinary professional services fee of CAD 167 for the spring visit.

The reported 2020 diagnostic testing costs established by Prairie Diagnostic Services’ fee guide were used (Prairie Diagnostic Services Inc., Saskatoon, SK, Canada). The ELISA test cost was CAD 13.50 for submissions with less than 90 samples and CAD 10.50 for submissions with 90 or more samples. The individual PCR test cost was CAD 46.00 for submissions with less than 100 samples and CAD 37.50 for submission with 100 or more samples. The PCR test on pooled samples was valued at CAD 62.50 for pools of five. No cost was included for veterinary consultation to establish the testing protocol or to interpret test results.

Variable expenses related to production arose from feed, veterinary costs, and labor. All variable expenses were derived from the 5-year average Canadian Cow–Calf Cost of Production results for 47 herds with 200–400 cows in Alberta, Saskatchewan, and Manitoba [17]. A five-year average of production costs per cow wintered was calculated for key expense categories, including purchased feed, machinery, fuel, land, veterinary care, and both paid and unpaid labor. These “$/cow wintered” costs represented total herd expenses attributed to the breeding cow herd. To accurately assign costs to mature breeding cows, adjustments were made based on herd composition, specifically, the proportion of breeding cows relative to heifer retention and the bull-to-cow ratio. This calculation yielded the cost to maintain approximately 1.25 animal units per year, equivalent to one mature cow (Supplementary Materials S2).

Expenses were only applied to post-weaned animals, as costs for suckling calves were included in the cow’s expenses. Costs for each livestock class were assumed proportional to a mature cow based on animal unit equivalents. Annual costs per head were distributed seasonally, with 70% of expenses incurred during the winter-feeding period and 30% during summer grazing. Total annual expenses were then calculated as the sum of costs across all livestock classes, including breeding stock expenses and additional costs associated with Johne’s disease sampling and testing.

#### 2.2.3. Net Present Value

As the goal was to estimate 10-year NPV, disease and economic parameters were projected for future years to reflect reasonable market conditions. The base partial budget output was used for year 1. To reflect market changes in future years (i.e., years 2 to 10), livestock market prices were adjusted from year 1 values proportional to the percentage change in the forecast values by the USDA Agriculture Projections Outlook [18]. The same trend was applied to all classes of cattle, as well as breeding stock expenses. All other costs were adjusted annually for inflation at a rate of 2%.

An NPV calculation was then performed by summing all future annual net cash flows (revenues minus costs (C)) divided by (1 + *r*)^*t*^, where *r* is the discount rate and *t* is the year of the cash flow, thereby converting future values into their present value equivalents. A 7% discount rate (*r*) was applied to reflect the time value of money. NPV was estimated for each iteration (*n* = 5000) for each test-and-cull scenario examined in this study (see Section 2.3, Section 2.5 and Section 2.6).

### 2.3. Test-and-Cull Strategies

Several potential test-and-cull strategies were created that were modeled to maintain or reduce the mean 10-year JD prevalence with the baseline model of internal replacement heifers and an external mature bred cow population with 1% JD prevalence. Each strategy was a unique combination of diagnostic test, sampling frequency, risk-based sampling, and sample pooling. To illustrate the potential impact of each strategy in the long term, it was assumed that each single strategy would be applied for the full 10 years (i.e., a producer picked that strategy and consistently implemented it for 10 years regardless of JD prevalence or NPV).

The diagnostic test and sample combinations considered to create each of the unique test-and-cull strategies included serum from individual animals tested with ELISA, feces from individual animals tested with PCR, and pools of feces from five animals tested with PCR with follow-up PCR testing on feces from individual animals contributing to positive sample pools. The testing frequencies considered were every 6, 12, or 24 mo. All strategies sampled cows in the fall concurrent with pregnancy diagnosis, while the semi-annual approach also tested cows prior to moving to the summer breeding field. Risk-based sampling was considered by excluding mature animals with four previous negative tests from sampling.

A total of seven unique test-and-cull strategies were reported based on previous evidence of potential for disease control [14]: (1) individual fecal testing with PCR semi-annually (6 mo increments); (2) individual fecal testing with PCR annually (12 mo); (3) individual fecal testing with PCR biennially (24 mo); (4) individual fecal testing with PCR in animals that had fewer than 4 prior negative PCR tests annually (12 mo); (5) pooled fecal testing with PCR semi-annually (6 mo); (6) pooled fecal testing with PCR annually (12 mo); and (7) individual serum ELISA tests semi-annually (6 mo).

### 2.4. Determining the “Choice Set” of Strategies

The determination of which JD test-and-cull strategy, or sets of strategies, are advisable requires a trade-off between short-term profits and long-term prevalence. All producers will seek to maximize their utility, or the perceived value with respect to testing and culling, but individual producers may rationally select different strategies that emphasize one outcome over the other. To account for some of these differences and explore the potential options depending on preference, several sensitivity analysis scenarios were developed that considered different JD prevalence, replacement female management choices, and livestock market and cost of production considerations (described in Section 2.5). For each given scenario, test-and-cull strategies were ranked by the key outcome factors that would influence a producer’s decision: final JD prevalence (the option that reduces the disease as much as possible), NPV (the option that achieves the best economic benefit), and sum of rank for prevalence and NPV (the option(s) that aim to balance disease reduction while achieving best possible economic benefit). A “choice set” of strategies for each scenario was then identified, which reflects the optimal set of recommendations that a producer might reasonably want to choose from. The choice set included the best ranked option for prevalence, the best ranked option for NPV, and the top-ranking option from the ranked sum for prevalence and NPV. The resulting set thus provided options that could be recommended to any producer, which would enable them to choose based on the outcome(s) they preferred to prioritize (i.e., minimize JD prevalence, maximize NPV, or an optimal balance of both).

### 2.5. Sensitivity Analysis

There are several key factors that may influence a producer’s choice over which test-and-cull strategy is preferred. Firstly, producers might reasonably be expected to differ in their preference to raise internal replacement heifers versus purchasing external stock. Secondly, the JD prevalence in the external mature bred female population is not known with certainty, and the true prevalence would certainly influence the ultimate outcome. Finally, the economics of JD control may be affected by livestock market values and cost of production. These three factors were considered through sensitivity analysis, each of which are described further below.

#### 2.5.1. Replacement Female Management Choices

Instead of raising replacements, an external replacement option was considered. The external option sold all female calves at weaning. All replacement females were purchased as mature, bred cows. This scenario was estimated for situations when the JD prevalence in the external bred cow population was 1% and 10%.

#### 2.5.2. JD Prevalence

The baseline test-and-cull strategy assumed a 1% prevalence of JD in the external mature bred cow population. The effect of this assumption was tested by considering 10% JD prevalence in the external population.

#### 2.5.3. Livestock Market and Cost of Production

The NPV was estimated with a relatively strong and weak livestock market by exploring the sensitivity of an increase or decrease in the values for livestock purchased and sold by 10%, respectively, while expenses were held at baseline. The effect of the livestock market on the choice set was explored with the baseline scenario of internal replacement heifers and 1% JD prevalence in the external mature cow population.

The NPV was also estimated with a high and a low cost of production by exploring the sensitivity of the model using the minimum and maximum reported cost of production values, while livestock values were held at baseline.

### 2.6. Relative Value of Preventing JD

The primary objective of this study was to assist producers with JD in selecting a test-and-cull strategy. A secondary objective was to understand the relative cost of JD to the industry and the value of ensuring herds remain JD free. A set of six scenarios were examined, all with 0% initial JD and no testing. Three scenarios used internal replacement heifers with 0%, 1%, or 10% JD prevalence in the external population. The other three scenarios use external mature bred cows with 0%, 1%, or 10% JD prevalence. The relative difference between these scenarios was then used to provide an estimate of the cost of JD.

### 2.7. Statistical Analysis

All analyses were completed in a commercial software program (Stata/SE16, StataCorp LLC, College Station, TX, USA). Regression models were used to summarize and compare stochastic iterations (*n* = 5000) among each set of scenarios (see Section 2.3, Section 2.5 and Section 2.6) and generate predicted means and 95% confidence intervals (95% CI) with a Scheffe’s adjustment for multiple comparisons.

## 3. Results

### 3.1. Effect of JD Prevalence with Internal Replacements

#### 3.1.1. Baseline JD Prevalence with Internal Replacements

The JD prevalence rose from 6% to an average of 17% after 10 years when no testing was performed under the baseline scenario where internal replacements were used and the expected JD prevalence in purchased stock was 1% (Table 1).

All seven test-and-cull strategies resulted in lower prevalence, with six of the seven resulting in higher NPV at 10 years compared to no testing (Table 1). The choice set included only strategies that tested feces from individual animals with PCR. The most effective strategy to reduce JD tested feces from individual animals with PCR semi-annually, but this was the least profitable option based on NPV; even less profitable than not testing under the baseline scenario. The most profitable strategy tested feces from individual animals with PCR biennially (24 mo) but ranked sixth highest among all test-and-cull strategies in final JD prevalence (Table 1). The best combination of controlling prevalence and optimizing NPV in the baseline scenario resulted from testing the feces from individual cows with PCR annually or testing individual cows with PCR annually that had less than four prior negative PCR tests (Table 1).

#### 3.1.2. Internal Replacements When External JD Prevalence 10%

A JD prevalence of 10% in the external population available for purchase resulted in significantly higher final JD prevalence and lower NPV for all test-and-cull strategies compared to an external population prevalence of 1% (Table 2 (A)). The relative effect of test-and-cull strategies on prevalence and profits, compared to a lower baseline JD prevalence, however, was similar. As was the choice set; the choice set included strategies that tested feces from individual animals with PCR. Three of the four strategies in the choice set when the external mature bred cow population had 10% JD prevalence (individual fecal sampling with PCR at 6 mo, at 12 mo, and at 24 mo; Table 2 (A)) were the same as the choice set when the external mature bred cow population had 1% JD prevalence (Table 1).

### 3.2. Effect of Management Choice to Purchase Replacement Females

#### 3.2.1. Baseline JD Prevalence with Purchased Replacements When External JD Prevalence 1%

No testing was the most profitable strategy with external replacements and an external JD prevalence in the population available for purchase of 1% (Table 2 (B)). This resulted in 1.6% (95% CI; 1.5–1.6) final JD prevalence. While this is considerably lower than the starting prevalence, all test-and-cull strategies resulted in lower final 10-year prevalence than no testing (Table 2 (B)). Again, the most effective strategy for reducing JD prevalence was individual PCR testing every 6 mo. PCR testing on pools of feces from five animals every 12 mo was identified as the optimal strategy to balance JD prevalence and NPV (Table 2 (B)). Individual ELISA testing, PCR testing pools of feces from five animals every 6 mo, and individual fecal sampling using PCR at 12 mo were ranked as just slightly less effective (Table 2 (B)).

#### 3.2.2. Baseline JD Prevalence with Purchased Replacements When External JD Prevalence Is 10%

Similar to the use of internal replacements and a 1% JD prevalence in purchased stock (Table 1), the choice set included strategies that tested feces from individual animals with PCR when purchased replacements were used and an external JD prevalence of 10% was assumed (Table 2 (C)). The most effective strategy to reduce JD tested feces from individual animals with PCR semi-annually, but this was the least profitable option based on NPV (Table 2 (C)) and far less profitable than not testing. The most profitable strategy tested feces from individual animals with PCR biennially (24 mo) but ranked seventh highest among all test-and-cull strategies in final JD prevalence (Table 2 (C)). It is noteworthy that no testing produced the second most profitable outcome. The best combination of controlling prevalence and optimizing NPV resulted from testing the feces from individual cows with PCR annually (Table 2 (C)).

### 3.3. Sensitivity to Livestock Market Price and Cost of Production

The strength of the livestock market did not alter the pattern or choice set for the baseline scenario of internal replacement heifers and a 1% JD prevalence (Table 3).

While cost of production (COP) had little effect on the preferred strategy (Table 3), changes in COP had a much larger impact on net present value (NPV) than equivalent shifts in market prices. Because JD testing and culling affect only a small proportion of animals each year, even sizable market swings had minimal influence on overall profitability, underscoring the importance of using accurate, herd-specific COP data. Overall, variation in COP and market conditions exerted far greater influence on NPV than the differences among test-and-cull strategies, highlighting that the financial risk incurred from investing in an ongoing JD control protocol is small relative to the variability in both markets and COP.

### 3.4. Relative Value of Preventing JD

The final 10-year prevalence of JD in herds with no testing, 0% initial JD, and an external JD prevalence of 1.0% was similar regardless of the replacement female management choice (Table 4). However, when animals were sourced from a population with 10% JD, the prevalence rose to 6.2% with internal replacement heifers and 7.2% with external mature bred cows after 10 years.

The NPV for herds with 0% JD and purchasing animals from a population with either 0% or 1% JD were >CAD 600,000, which was the highest for any situation considered. Only the scenario when external mature cows were purchased from a population with 10% JD (Table 4) had an NPV lower than any of the situations with 6% initial JD and a test-and-control strategy (Table 1 and Table 2).

The optimal scenario modeled was 0% initial JD prevalence and the ability to purchase animals from a population with 0% JD. This was included to provide a comparison of the effect of JD and to illustrate how the model assumptions influenced the relative profitability for each replacement female choice.

### 3.5. Best Performing Test-and-Cull Strategies Across Scenarios

Table 5 summarizes which test-and-cull strategies performed best across all modeled herd and market scenarios, indicating for each combination of replacement management and external JD prevalence which strategy minimized 10-year JD prevalence, maximized NPV, or offered the best balance of both. In short, annual individual PCR testing (with or without the four-test exclusion rule) consistently achieved the optimal balance between profitability and disease control, while semi-annual testing minimized prevalence and biennial testing maximized NPV.

## 4. Discussion

This study used an updated version of an ABM [14] integrated with a partial budget framework to assess the epidemiological and economic performance of test-and-cull strategies for JD in Western Canadian beef herds. The ABM structure was particularly valuable for capturing the biological complexity, stochasticity, and long-time horizon inherent to JD transmission. Unlike deterministic models, the ABM simulated individual animal interactions and dynamic replacement patterns, accounting for uncertainty in infection status, diagnostic accuracy, and management responses over multiple years. This level of granularity allowed the model to generate more realistic projections of both prevalence and profitability under variable herd and market conditions.

The use of NPV as the primary economic outcome appropriately reflects the long-term nature of JD control decisions by accounting for the time value of money and discounting future costs and revenues. Unlike short-term metrics such as return on investment or annual cash flow, NPV captures the delayed and cumulative benefits of disease control. The 7% discount rate used aligns with previous Johne’s disease (JD) economic studies and reflects a realistic value for moderate-risk beef operations in Canada. This rate represents a midpoint within the typical 5–10% range commonly applied in livestock investment analyses and was selected to maintain consistency with prior JD economic modeling work [3,6].

Under baseline conditions representing a typical 300-cow Western Canadian herd with internal replacements and a 1% JD prevalence in purchased stock, the ABM predicted that JD prevalence could rise substantially to an average of 17% over a 10-year horizon in the absence of intervention. All test-and-cull strategies evaluated reduced prevalence, and six of the seven increased herd profitability compared with not testing. This demonstrates that proactive JD control is likely to be both biologically effective and economically defensible under most commercial scenarios, a finding demonstrated in similar economic modeling studies [3,5].

To guide practical decision-making, a “choice set” of strategies was defined that captured producer preferences for either minimizing JD prevalence, maximizing profitability, or achieving a balance of both. The results were consistent across management and market scenarios and clearly reflected different producer priorities. Semi-annual individual PCR testing (Individuals–PCR–6) produced the lowest 10-year JD prevalence but was the least profitable approach. Biennial PCR testing (Individuals–PCR–24) yielded the highest NPV but achieved more modest prevalence reductions. Annual individual PCR testing (Individuals–PCR–12) and annual testing with exclusion of cows having four prior negative results (Individuals–PCR–12 Max 4) consistently balanced disease reduction with profitability, ranking among the top options across scenarios. Our findings demonstrate that a single “best” JD control strategy does not exist; rather, there are multiple profitable pathways depending on a producer’s management goals and cash-flow tolerance. For example, a risk-averse producer prioritizing long-term herd health may prefer annual PCR testing, while one focused on near-term profitability could opt for biennial testing without substantially compromising control. The consistency of the choice set across scenarios strengthens confidence in the robustness of these recommendations.

Management of replacement females had a strong influence on the economics of JD control. When herds purchased mature bred cows rather than raising internal replacements, no-testing scenarios occasionally appeared economically competitive, particularly when JD prevalence in the external population was low (1%). However, this apparent advantage was short-sighted, as increases in the JD prevalence of purchased stock (10%) led to rapid increases in within-herd prevalence and declining NPV. Under such higher-risk conditions, the profitability of no testing diminished, and test-and-cull strategies regained clear advantage. This pattern highlights that the epidemiological context of animal sourcing, specifically the infection risk of incoming stock, is a decisive factor shaping the perceived economic value of testing.

These findings provide nuance to the conclusions by previous authors that the cost-effectiveness of test-and-cull strategies depends almost entirely on the implementation of management changes to minimize calf exposure and environmental contamination [3,5]. While the ABM used in the present study did not enable our ability to explore the impact of changes to management practices, our findings clearly demonstrate that the cost-effectiveness of solely a test-and-cull strategy depends strongly on the epidemiological context, particularly the starting herd prevalence and diagnostic test performance. Our study demonstrates that the viability of test-and-cull strategies, compared to no action, increases as prevalence increases. This reinforces that the perceived advantage of foregoing testing in low-risk sourcing scenarios may be short-lived; as external infection pressure increases, the economic justification for proactive test-and-cull programs strengthens.

Interestingly, regardless of whether internal or external replacements were used, the same general set of strategies remained optimal. Individual PCR testing every 12 mo continued to represent the best compromise between economic and epidemiologic outcomes, while biennial testing provided the most cost-efficient option. Semi-annual testing produced the lowest prevalence in all scenarios, yet the cost of such frequent sampling outweighed its benefits in commercial settings.

Sensitivity analyses exploring the effects of COP and livestock market fluctuations revealed that the overall profitability of JD control was more sensitive to production costs than to market prices. Increasing or reducing cattle prices by 10% produced only marginal shifts in NPV because JD control decisions affect a relatively small proportion of animals each year. In contrast, increases in COP substantially reduced profitability across all strategies. These findings are particularly relevant given the current economic environment. According to the 2024 Canfax Cost of Production Network, the average total cost of production for Canadian cow–calf operations rose to CAD 1850 per cow, a 5% increase over 2023, reflecting ongoing inflationary pressure and rising depreciation costs [19]. While cattle markets remained historically strong, average revenue reached CAD 2012 per cow, up 16% from 2023 due to record-high calf prices, input inflation continued to erode margins [19]. This economic context highlights that disease control investments compete against persistent cost pressures.

In our model, an annual inflation assumption of 2% was used to project future costs, which in hindsight underestimates recent conditions. Canadian inflation and interest rates have been markedly higher in 2023–2025, with sustained increases in input prices and borrowing costs [20]. These real-world pressures would likely compress the NPV of all long-term JD control strategies but would not change their relative ranking. The key implication is that, while JD control has economic importance, its effect on whole-farm profitability is modest relative to broader cost and price variability. Producers may therefore overestimate the financial risk of adopting testing programs; in reality, JD control costs are minor compared with annual fluctuations in feed or fuel expenses.

The analysis of JD-free herds provided additional perspective on the long-term value of maintaining disease freedom. When herds remained JD-free and sourced animals from low-risk populations, NPVs exceeded CAD 600,000, significantly higher than any scenario in which JD became established. Conversely, herds purchasing replacements from high-prevalence populations (10%) experienced both higher JD prevalence (6–7%) and lower NPVs (~CAD 440,000–570,000). These results emphasize that preventing disease introduction yields far greater economic benefits than attempting to control JD once endemic, which is supported by numerous other studies [1,3,5].

While this modeling approach provides valuable insights, several limitations must be acknowledged. First, the simulations represent a specific Western Canadian production system and should not be extrapolated to dairy contexts or to other geographic regions without modification. While there have been other agent-based models published in dairy herds [21,22,23,24], they are not directly comparable to the present study as they include management options that are not practiced in most North American beef herds such as raising calves separately from the cows and in-doors [24]. Second, each strategy was applied consistently over a fixed 10-year period, whereas, in reality, producers may adapt their management annually based on disease status or market shifts. Third, although the ABM incorporated fundamental management parameters, it did not explicitly model the effects of a specific set of best-management practices (e.g., calving area hygiene, manure handling, or biosecurity training) whose influence on JD prevalence remains poorly quantified. Empirical data on these factors would allow for future refinement of the model.

## 5. Conclusions

This study demonstrates that JD can be controlled economically in Western Canadian beef herds, while failure to implement control measures leads to steadily rising prevalence and long-term financial losses. Across the range of modeled scenarios, several test-and-cull strategies consistently reduced prevalence and improved profitability, underscoring their robustness as control options even under variable market conditions, herd management practices, and external disease risks. Importantly, no single strategy was universally optimal; instead, multiple approaches offered producers the flexibility to align JD control with their operational handling patterns and management preferences. Together, these findings highlight that proactive JD control is both feasible and economically justified and that producers can select from a set of robust, adaptable strategies to safeguard herd health and profitability.

## Figures and Tables

**Table 1:** Mean (95% CI) 10-year JD prevalence and NPV (hundred thousand CAD) resulting from no testing and the use of seven unique test-and-cull strategies for a scenario with 1% JD in purchased stock and only internal replacement, with ranks for most effective in reducing 10-year JD prevalence (Prevalence Rank), maximizing NPV (NPV Rank), and optimizing prevalence and NPV (Rank Sum).

Replacement Female Management and JD Prevalence in External Population	Test-and-Cull Strategy	10-Year JD Prevalence	Prevalence Rank	NPV (Hundred Thousand CAD)	NPV Rank	Rank Sum
		Mean (95% CI)		Mean (95% CI)		
Internal replacements and 1% JD in purchased stock	No testing	16.6 (16.6–16.7)	8	4.11 (4.09–4.14)	7	8
	**Individuals–PCR–6**	0.4 (0.3–0.5)	1 *	3.84 (3.81–3.87)	8	5
	**Individuals–PCR–12**	1.3 (1.2–1.3)	2	4.41 (4.38–4.44)	4	1 *
	**Individuals–PCR–24**	2.9 (2.8–3.0)	6	4.84 (4.81–4.87)	1 *	3
	**Individuals–PCR–12 Max 4**	2.3 (2.2–2.4)	4	4.48 (4.45–4.51)	2	1 *
	Pools of 5–PCR–6	1.9 (1.8–2.0)	3	4.3 (4.27–4.33)	6	5
	Pools of 5–PCR–12	4.6 (4.5–4.7)	7	4.36 (4.33–4.39)	5	7
	Individuals–ELISA–6	2.5 (2.4–2.5)	5	4.43 (4.40–4.46)	3	4

* Test-and-cull strategy offering the best outcome for that rank category. Bolded options ranked highest for at least one metric. Abbreviations: JD—Johne’s disease, NPV—net present value, CAD—Canadian dollar, PCR—polymerase chain reaction, ELISA—enzyme-linked immunosorbent assay, CI—confidence interval.

**Table 2:** Mean (95% CI) 10-year JD prevalence and NPV (hundred thousand CAD) resulting from no testing and the use of seven unique test-and-cull strategies scenarios with (**A**) 10% JD in purchased stock and only internal replacements, (**B**) 1% JD in purchased stock and buying external replacements, and (**C**) 10% JD in purchased stock and buying external replacements, with ranks for most effective in reducing 10-year JD prevalence (Prevalence Rank), maximizing NPV (NPV Rank), and optimizing prevalence and NPV (Rank Sum).

Replacement Female Management and JD Prevalence in External Population	Test-and-Cull Strategy	10-Year JD Prevalence		NPV (Hundred Thousand CAD)		
		Mean (95% CI)	Prevalence Rank	Mean (95% CI)	NPV Rank	Rank Sum
(A) Internal replacements and 10% JD in purchased stock	No testing	21 (20.9–21.1)	8	3.78 (3.75–3.81)	8	8
	**Individuals–PCR–6**	1.0 (0.9–1.1)	1 *	3.70 (3.67–3.73)	8	4
	**Individuals–PCR–12**	2.6 (2.5–2.6)	2	4.16 (4.13–4.19)	3	1 *
	**Individuals–PCR–24**	6.8 (6.7–6.9)	6	4.45 (4.42–4.48)	1 *	3
	Individuals–PCR–12 Max 4	4.5 (4.5–4.6)	5	4.13 (4.10–4.16)	4	4
	Pools of 5–PCR–6	3.6 (3.5–3.6)	3	4.00 (3.97–4.03)	6	4
	Pools of 5–PCR–12	7.2 (7.1–7.3)	7	4.02 (3.99–4.05)	5	7
	Individuals–ELISA–6	4.3 (4.3–4.4)	4	4.2 (4.18–4.23)	2	2
(B) External replacements and 1% JD in purchased stock	**No testing**	1.6 (1.5–1.6)	8	4.27 (4.24–4.30)	1 *	5
	**Individuals–PCR–6**	0.3 (0.2–0.3)	1 *	3.05 (3.02–3.08)	8	5
	Individuals–PCR–12	0.3 (0.3–0.4)	2	3.79 (3.76–3.82)	7	2
	Individuals–PCR–24	0.6 (0.5–0.7)	7	4.15 (4.12–4.18)	2	5
	Individuals–PCR–12 Max 4	0.5 (0.4–0.6)	5	3.87 (3.84–3.89)	6	8
	Pools of 5–PCR–6	0.4 (0.3–0.5)	3	3.87 (3.84–3.90)	5	2
	**Pools of 5–PCR–12**	0.5 (0.5–0.6)	6	4.13 (4.10–4.16)	3	1 *
	Individuals–ELISA–6	0.5 (0.4–0.5)	4	3.89 (3.86–3.91)	4	2
(C) External replacements and 10% JD in purchased stock	No testing	8.2 (8.1–8.2)	8	3.52 (3.49–3.55)	2	7
	**Individuals–PCR–6**	2.5 (2.4–2.6)	1 *	2.57 (2.54–2.60)	8	3
	**Individuals–PCR–12**	2.8 (2.7–2.9)	2	3.24 (3.21–3.27)	6	1 *
	**Individuals–PCR–24**	5.0 (4.9–5.0)	7	3.53 (3.50–3.56)	1 *	1
	Individuals–PCR–12 Max 4	3.6 (3.5–3.7)	4	3.26 (3.23–3.29)	5	3
	Pools of 5–PCR–6	3.3 (3.2–3.4)	3	3.18 (3.15–3.20)	7	7
	Pools of 5–PCR–12	4.2 (4.1–4.3)	6	3.41 (3.38–3.44)	3	3
	Individuals–ELISA–6	3.7 (3.6–3.8)	5	3.31 (3.29–3.34)	4	3

* Test-and-cull strategy offering the best outcome for that rank category. Bolded options ranked highest for at least one metric. Abbreviations: JD—Johne’s disease, NPV—net present value, CAD—Canadian dollar, PCR—polymerase chain reaction, ELISA—enzyme-linked immunosorbent assay, CI—confidence interval.

**Table 3:** Mean (95% CI) 10-year JD prevalence and NPV (hundred thousand CAD) resulting from no testing and the use of seven unique test-and-cull strategies scenarios when high and low cost of production and strong and weak livestock market assumptions are modeled.

Replacement Female Management and JD Prevalence in External Population		10-Year JD Prevalence			NPV (Hundred Thousand) Mean (95% CI)	
	Test-and-Cull Strategy	Mean (95% CI)	Low Cost of Production	High Cost of Production	Strong Livestock Markets	Weak Livestock Markets
Internal replacements and 1% JD in purchased stock	No testing	16.6 (16.6–16.7)	8.70 (8.65–8.75)	−8.72 (−8.75–−8.69)	6.40 (6.36–6.44)	1.63 (1.61–1.65)
	Individuals–PCR–6	0.4 (0.3–0.5)	8.47 (8.42–8.52)	−9.02 (−9.05–−8.98)	6.29 (6.25–6.33)	1.20 (1.19–1.22)
	Individuals-PCR–12	1.3 (1.2–1.3)	9.06 (9.01–9.11)	−8.51 (−8.54–−8.48)	6.85 (6.81–6.89)	1.78 (1.76–1.80)
	Individuals–PCR–24	2.9 (2.8–3.0)	9.55 (9.50–9.60)	−8.13 (−8.16–−8.10)	7.35 (7.31–7.39)	2.14 (2.12–2.16)
	Individuals–PCR–12 Max 4	2.3 (2.2–2.4)	9.12 (9.07–9.17)	−8.45 (−8.48–−8.42)	6.90 (6.86–6.94)	1.86 (1.84–1.88)
	Pools of 5–PCR–6	1.9 (1.8–2)	8.90 (8.86–8.95)	−8.55 (−8.59–−8.52)	6.71 (6.67–6.75)	1.69 (1.68–1.71)
	Pools of 5–PCR–12	4.6 (4.5–4.7)	8.98 (8.93–9.03)	−8.53 (−8.56–−8.50)	6.75 (6.71–6.79)	1.78 (1.76–1.79)
	Individuals–ELISA–6	2.5 (2.4–2.5)	9.04 (8.99–9.09)	−8.42 (−8.45–−8.39)	6.83 (6.79–6.87)	1.84 (1.82–1.85)

Abbreviations: JD—Johne’s disease, NPV—net present value, CAD—Canadian dollar, PCR—polymerase chain reaction, ELISA—enzyme-linked immunosorbent assay, CI—confidence interval.

**Table 4:** Mean (95% CI) 10-year JD prevalence and NPV (hundred thousand CAD) resulting from no testing and a combination of different replacement female management and JD prevalences in the external population.

Replacement Female Management and JD Prevalence in External Population	Test-and-Cull Strategy	10-Year JD Prevalence	NPV (Hundred Thousand CAD)
		Mean (95% CI)	Mean (95% CI)
Internal replacements and 0% JD in purchased stock	No testing	0.0 (−0.1–0.1)	6.07 (6.06–6.08)
Internal replacements and 1% JD in purchased stock	No testing	0.7 (0.6–0.7)	6.03 (6.00–6.06)
Internal replacements and 10% JD in purchased stock	No testing	6.2 (6.1–6.3)	5.72 (5.69–5.75)
External replacements and 0% JD in purchased stock	No testing	0.0 (−0.1–0.1)	5.16 (5.13–5.19)
External replacements and 1% JD in purchased stock	No testing	0.7 (0.6–0.8)	5.09 (5.06–5.12)
External replacements and 10% JD in purchased stock	No testing	7.2 (7.2–7.3)	4.41 (4.38–4.44)

Abbreviations: JD—Johne’s disease, NPV—net present value, CAD—Canadian dollar, CI—confidence interval.

**Table 5:** Visualization of the test-and-cull strategies that were identified as being ranked as most effective in reducing 10-year JD prevalence (Prevalence Rank = ⮾), maximizing NPV (NPV Rank = $), and optimizing prevalence and NPV (Rank Sum = ☑) for each combination of different replacement female management and JD prevalence in the external population depicted in Table 1 and Table 2.

Test-and-Cull Strategy	Replacement Female Management and JD Prevalence in External Population			
	Internal Replacements and 1% JD in Purchased Stock	Internal Replacements and 10% JD in Purchased Stock	External Replacements and 1% JD in Purchased Stock	External Replacements and 10% JD in Purchased Stock
No testing			$	
Individuals–PCR–6	⮾	⮾	⮾	⮾
Individuals–PCR–12	☑	☑		☑
Individuals–PCR–24	$	$		$
Individuals–PCR–12 Max 4	☑			
Pools of 5–PCR–6				
Pools of 5–PCR–12			☑	
Individuals–ELISA–6				

Abbreviations: JD—Johne’s disease, NPV—net present value, CAD—Canadian dollar, PCR—polymerase chain reaction, ELISA—enzyme-linked immunosorbent assay.

## Data Availability

The original contributions presented in this study are included in the article/Supplementary Materials. Further inquiries can be directed to the corresponding author.

## Supplementary Materials

The following supporting information can be downloaded at: https://www.mdpi.com/article/10.3390/vetsci12121210/s1, File S1: Agent-Based Model Documentation; File S2: Economics Supplement; File S3: Supplemental Economic Tables.

## Abbreviations

The following abbreviations are used in this manuscript:

ABM	Agent-Based Model
BCS	Body Condition Score
CAD	Canadian Dollar
CI	Confidence Interval
COP	Cost of Production
ELISA	Enzyme-Linked Immunosorbent Assay
JD	Johne’s Disease
MAP	*Mycobacterium avium* spp. *paratuberculosis*
NPV	Net Present Value
ODD	Overview, Design concepts, and Details
PCR	Polymerase Chain Reaction
PDS	Prairie Diagnostic Services
SCIC	Saskatchewan Crop Insurance Corporation
USDA	United States Department of Agriculture
