# Supplementary material for: Johne’s Disease Control in Beef Cattle: Balancing Test-and-Cull Strategies with Economic and Epidemiological Trade-Offs
Source: Vet Sci. 2025 Dec 17;12(12):1210. doi: 10.3390/vetsci12121210 (PMC12737406; doi:10.3390/vetsci12121210)
Supplement: Supplementary file 1 [file vetsci-12-01210-s001.zip › SupplementalFile2_EconomicsSupplement_16Nov2025.pdf]

Supplementary Material File 2

Johne's Disease Control in Beef Cattle: Balancing Test-and-Cull Strategies with Economic and Epidemiological Trade-offs

Leigh Rosengren, Steven M. Roche, Kathy Larson, Cheryl L. Waldner*

1 Supplementary Data for Economics Analysis

This supplement provides additional detail for "**Johne's Disease Control in Beef Cattle: Balancing Test-and-Cull Strategies with Economic and Epidemiological Trade-offs**" regarding the methods and assumptions made to estimate expenses and incomes.

1.1 Additional Details for Partial Budget and Net Present Value Calculations

1.1.1 Income

Cattle were classified into livestock classes based on gender, age (months), and production purpose (**Table S1, Supplemental File 3 - Excel**). Revenues were calculated for each livestock class as the product of weight and market price with the exception of bred heifers which were calculated as the product of the number of head sold by price per head. Annual revenues were the sum of revenues from all livestock classes. Livestock market values were representative of Saskatchewan, Canada between 2017 and 2021 (Prices | SCIC, https://www.scic.ca/agristability/prices/, Access 01 November 2025).

The reported prices for October, November and December in 2017 – 2021 were averaged (**Table S2**). These months corresponded to the seasonal marketing of cattle in Western Canada and the production cycle modelled in the ABM and the years to the 5-year period reported for cost of production. Steer and heifer calves used the 501 – 600 lbs (227 – 272 kg) weight class values to represent a 550 lbs (249 kg) animal. The calf price, for each gender, was adjusted using a $0.10/CWT slide applied to the average weight reported from the population model for a 550 lbs (249 kg) base weight. No open heifers between 8 – 12 months were sold in the model.

Slaughter bull values as provided on a monthly basis for October through December of 2017 to 2021 were averaged for Saskatchewan sales (**Tables S1 and S2, Supplemental File 3 - Excel**) (Canfax Research Services, Personal Communication Huiting Huang, 2023). The same price was attributed to yearling and mature bulls.

Yearling heifers sold due to non-pregnancy were allocated the 901 – 1000 lbs yearling heifer price (**Tables S1 and S2, Supplemental File 3 - Excel**).

Mature cows were marketed in one of four conditions based on being young or old and in good or poor body condition (**Tables S1 and S2, Supplemental File 3 - Excel**). Canada uses a five-point body condition score (BCS) ranking 0 - 2.0 as underconditioned, 2.5 in adequate, 3.0-3.5 in good,

and >3.5 as over-conditioned (Beef Cattle Research Council). Cows less than 10 years were classified as young. Young cows with a BCS ≥2.5 were allocated a D1/D2 market value (Saskatchewan's Dashboard - D1/D2 Cows – Saskatchewan, https://dashboard.saskatchewan.ca/agriculture/livestock-prices/cows, Accessed 01 November 2025). Young cows with a BCS <2.5 were allocated a D3 market value. All cows older than 10 years, regardless of BCS, were allocated a D3 market value. Regardless of age, 80% of thin cows were assumed to be 200 lbs (91 kg) lighter than cows in good condition while 20% were considered to be so thin they were unsuitable to market and were allocated 0$ for market value.

Bred heifers were the only class of breeding stock sold. This occurred when the number of bred heifers exceeded the number required to maintain the herd at 300 head (Prices | SCIC, https://www.scic.ca/agristability/prices/, Accessed 01 November 2025).

1.1.2 Expenses

Expenses arose from three categories: breeding stock purchases, JD sampling and testing, and variable inputs from feed, veterinary and labour.

Breeding stock purchases: Three classes of livestock were purchased. Market values were calculated from the average reported market prices for October, November, and December in 2017 – 2021 in Saskatchewan. The bred heifer and breeding cow prices were reported by SCIC (Prices | SCIC https://www.scic.ca/agristability/prices/ , Accessed 01 November 2025) (**Tables S1 and S2, Supplemental File 3 - Excel**). Breeding bull values were obtained from Canfax Research Services (personal communication, Huitang Huang).

JD sampling and testing: The cost of JD testing was estimated for sample collection and diagnostic test (**Table S3, Supplemental File 3 – Excel**; Johnson et al., 2022c). A $5 per head sample collection fee was set for blood collection and $2.50 per head for fecal sample collection. This fee was assumed to cover professional time to collect, prepare and ship samples as well as consumables used in sample collection. Courier costs to submit samples were estimated at $25 per visit. Strategies with annual sampling in the fall did not include fees for mileage or professional veterinary services beyond sample collection as it was assumed the veterinarian was attending the herd for pregnancy detection. Strategies with semi-annual sampling included a minimal mileage fee and veterinary professional services fee for the spring visit.

Variable inputs from feed, veterinary costs and labor: Variable expenses related to production arose from feed, veterinary costs, and labour (**Table S4, Supplemental File 3 - Excel**). All variable expenses were based on the Canadian Cow-Calf Cost of Production Network results. Results were reported by focus groups, each of which represents 3-6 producers of similar size and practices within a province. Focus group data were used for herds with 200 to 400 head located in Alberta, Saskatchewan and Manitoba. The geographical scope was expanded beyond Saskatchewan to include all of western Canada due to the limited number of participants in Saskatchewan. The data represented 47 herds from the following 12 focus groups: AB1, AB2, AB5, AB9, SK1a, SK1b, SK3, SK7, MB1, MB2, MB3a, MB3b. These herds reported a median breeding cow population of 275 (Range; 212-376), a median retained heifer rate of 13% (Range; 8 – 19%), and a median cow to bull ratio of 25 (Range; 21-27) which was reflective of the parameters in the ABM.

Details on the method to collect cost of production data are reported by Canfax (CanFax, 2022; Accessed 04 November 2025). The average of the 12 focus groups was used (**Table S5, Supplemental File 3 - Excel**). For sensitivity analysis the focus group with the lowest and highest reported cost of production were used respectively.

The 5-year average costs reported per cow wintered were totaled for purchased feed, machinery, fuel, land costs, veterinary care, paid and unpaid labour for each focus group (**Tables S4 and S5, Supplemental File 3 - Excel**). This '$/cow wintered' cost allocated all expenses to the breeding cow herd. The proportion of the herd attributable to breeding cows was calculated based on the percent heifer retention and bull to cow ratio (EQUATION).

$$Proportion\ of\ \$/Cow\ Wintered\ attributable\ to\ Mature\ breeding\ cows = {}^{1}\!/\!_{((Bull\ to\ cow\ ratio)\ +\ (\%\ heifer\ retention)\ +\ 1)}$$

The $/cow wintered were adjusted based on this proportion with the result considered the cost to maintain 1.25 animal units per year (i.e. a mature cow). (**Table S4, Supplemental File 3 - Excel)**

Only post-weaned animals accrued expenses; all costs for suckling calves were attributed to the cow. The expenses incurred by each livestock class were assumed proportional to a mature cow based on animal units. The annual cost per head was divided seasonally with 70% of costs accrued in winter and 30% in summer.

$$\boldsymbol{Annual\ Expenses = \sum livestock\ class \left[[0.7 * winter\ population * AU * cost\ per\ head] + [0.3 * summer\ population * AU * cost\ per\ head]\right]}$$ **+ Breeding stock expenses + JD sample and test costs**
